# Participating in university entrance exam despite repeated failure: a qualitative study of participants’ experiences

**DOI:** 10.5116/ijme.57eb.cc09

**Published:** 2016-10-22

**Authors:** Ruohollah Seddigh, Esmat Abdollahpour, Somayeh Azarnik, Behnam Shariati, Amir-Abbas Keshavarz-Akhlaghi

**Affiliations:** 1 Mental Health Research Center, Iran University of Medical Sciences, Tehran, Iran; 2 Department of Nephrology, Amir-Alam Hospital, Tehran University of Medical Sciences, Tehran, Iran

**Keywords:** College admission test, competitive behavior, student assessment, internship and residency, stress, Iran

## Abstract

**Objectives:**

To explore the experiences of general practitioners who continue to sit a highly-competitive residency entrance examination, despite repeated failure.

**Methods:**

This qualitative, exploratory study employed semi-structured, in-depth interviews with 35 candidates of a residency entrance exam who had failed the examination at least twice and were preparing to sit it again. Interview questions addressed the challenges they faced and how they managed these challenges. Interview data were audiotaped, transcribed, and analysed to identify themes.

**Results:**

The results demonstrated that more than 50% (n=19) of candidates struggled continuously and internally with different aspects of the exam. These include being under a great deal of pressure to succeed, failing to prioritize study materials, inefficient review during the final days  of preparation, and sitting the exam with an afflicted body. Furthermore, during the examination, they frequently used inefficient strategies to answer questions. Afterwards, they experienced feelings of freedom associated with having finished the exam.

**Conclusions:**

Participation in a highly-competitive examination exerts a considerable amount pressure on low-performing candidates. This climate not only results in burn out of participants, but it also influences their learning styles and identities as future physicians. It is therefore necessary to design a syllabus for both test candidates and policy makers, in order better to manage this environment. Candidates also should be aware of their individual weaknesses, in order to improve their studying skills.

## Introduction

The Residency Entrance Examination (REE) is a multiple-choice examination that employs negative marking for incorrect answers. It is held annually by the Ministry of Health and Medical Education (MHME) and is the only means of being accepted into medical specialty courses in Iran. According to the Education Department of the MHME, there were approximately 15500 exam candidates in 2013, 2000 of whom passed and were accepted. In each annual REE, more than 11000 candidates sit the exam for the second time or more.^1^ Although there are no formal studies on this topic, passing this exam is presumably one of the most significant challenges for general practitioners in Iran. Here, we use the term ‘general practitioner’ to refer to physicians who have only completed 18 months of basic post-graduate training (internship) in Iran and are yet a chance to be accepted into a specialty/residency training pathway (and do not currently hold board membership). Young general practitioners spend a great deal of time and effort becoming specialists. Despite these efforts, they must sometimes sit the REE several times. This is due to vast differences in the social and economic status of general practitioners and specialists in Iran. In this context, the highly competitive nature of the REE is comprehensible.

Participating in high-level competition induces somatic and cognitive anxiety,^2^ and negatively impacts self-confidence and some dimensions of performance.^3^^,^^4^ It also has deleterious effects on intrinsic motivation,^5^ feeling secure in the social environment,^6^ mood state, and sleep.^7^ However, positive expectations of goal achievement facilitate greater self-confidence and less anxiety during competition.^8^^,^^9^

Although competition is an important mediator, individual factors also play an important role in performance under such conditions. For example, medical students who fail their final exams use inappropriate learning strategies and maladaptive strategies to cope with and normalize their failure, and attribute it to external factors.^10^ Although they may also experience complex problems, such as academic, relationship, and mental health problems, they focus only on improving their examination performance and on maladaptive coping strategies.^11^ Studies also demonstrate that the level of motivation,^12^^-^^14^ the degree of concentration while testing^15^^,^^16^  and test-taking skills^17^^-^^19^ are positively related to, and anxiety negatively related to, examination success.^20^^-^^22^ The quality of the examination can also affect candidates' success.^23^

There is a paucity of research on the cognitive, emotional, and behavioral effects of long-term exposure to the stress of a highly-competitive academic examination, and on the ways in which candidates cope with them. In fact, most research in the field of competition relates to sports medicine. The aim of the current study was to gain insight into the broad range of factors associated with preparing for and retaking the REE, despite repeated failure. The main research question was, “Which cognitive, behavioral, and emotional strategies did candidates use to prepare themselves for the exam?” Based on the literature, we hypothesized that candidates with repeated failures would be under great pressure and would be more inclined toward using maladaptive strategies to pass the examination. Identifying these experiences in detail is critical to designing new studies and to developing interventions for this group of professionals.

## Methods

### Study design and participants

This study was approved by the Ethics Committee of the School of Behavioral Sciences and Mental Health of the Iran University of Medical Sciences. All participants gave informed consent to participate in the study. They were informed in writing that participation in this study would not affect any exams they may undertake in the future. No personal questions, related, for example, to personal relationships or political views were asked. Participation was voluntary and anonymous.

Interview data was qualitatively analyzed. Purposive sampling was used to select participants. Interviews took place at a university hospital library in the center of Tehran, Iran, where entrance is free for all physicians and where most visitors study for the REE or medical specialty board examination. We asked visitors about their reason for visiting the library and selected candidates who were about to study for REE and had failed the exam at least twice before. The initial interview screened for candidates with major depressive disorders, obsessive-compulsive disorder, generalized anxiety disorder, bipolar disorder, and psychotic disorders. These individuals were excluded from the study, as some of these psychiatric disorders negatively affect academic success.^24^ In the second interview, the interviewer focused on the study questions. Sampling continued until saturation point was reached (no more new themes were coded after five interviews). We conducted interviews a minimum of one to two months after the previous year's exam and one to two months before the next year's exam. None of the candidates declined participation in the study, which may be attributable to the empathetic attitude of the interviewer and to the desire, according to eight participants, to speak about one of the most difficult experiences of their lives.

A total of 35 general practitioners participated in the study (15 male, 20 female), 60% of whom had failed the REE at least twice and 40% of whom had failed it at least three times (with a maximum of five failures). All study participants were preparing to retake the exam the following year. Their average age was 32 years (range: 29 to 40 years). Twenty-one candidates were also married.

### Date collection method

Data were collected through semi-structured, in-depth interviews and recorded using a digital voice recorder. The interviewer was the top-placed candidate in Iran’s psychiatry final board examination in 2012. He did not have any connection with the study participants. The questions started with demographic information relevant to the study. The following main questions were then posed: (1) What passes through your mind when you think about last year's failure and how do you feel? (2) What pressures did you endure for these exams? (3) How did you study for this exam? and (4) How did you feel on exam day the previous time(s)?

Because of the limited number of studies about repeated failures in competitive examinations, the first three interviews aimed to answer the main questions and allowed participants to initiate topics and change the direction of the interviews.^25^ After these three initial interviews, one main question was added to the list: “What method do you use to answer the exam questions?”

We performed each interview after analysis of the previous interview throughout the study. In this way, the interviewer identified areas for improvement and identified clarifying questions (consistent with the main questions) for subsequent interviews. A single word code was used to identify each new piece of content. This was repeated until no new codes were identified after five continuous interviews. The average time for each interview was approximately 80 minutes (range: 60 to 120 minutes). In ten cases, a follow-up interview was conducted owing to ambiguity and disagreement among researchers on the meaning of interview data. Each follow-up interview lasted between 30 and 45 minutes. The study was conducted over nine months, from July 1st, 2014 to March 31st, 2015.

**Table 1:** Categories and subcategories of participants’ experiences

Categories	Subcategories
Inner struggle	Low self-confidence
	Overestimating others’ ability
	Magnifying exam difficulty
	Catastrophizing failure
	Pessimism towards test designers
	What will happen?
	Sweet daydreaming
Putting themselves under pressure	Studying long hours
	Studying hard
	Asceticism
	Doping
Inefficient mapping	Homogenization
	Excessive attention to detail
	Search about out of mind points
Improper final review	Wasted review
	Excessive review
Afflicted body	Anxiety
	Fatigue
	Weight gain
Inefficient test-taking skills	Overcomplicating
	Hastiness
	Answering in order
	Poor time management
	Hesitation at the end of the exam
	Obsessive checking
Liberation	Happy Liberation
	Bitter Liberation

### Data analysis

We analyzed our data using qualitative content analysis.^26^ First, recorded interviews were transcribed and studied by all authors independently. During this stage, authors read and reread the transcripts to understand and familiarize themselves with the data. Then, sentences related to research questions were selected and coded. A new code word was assigned if new content was discovered. Next, the contents were discussed in a shared meeting, and, if consensus was reached, code words were entered into the data gathering table. Data answered by more than 50% of participants were reported in the final report. The final results were categorized into 7 categories and 27 subcategories. 

## Results

Table 1 presents participant experiences according to these categories and sub-categories.

### Inner struggle

This theme reveals the cognitive status of candidates during the months prior to the exam. The first two features of this category were low self-confidence and overestimating others’ ability. Candidates evaluated their chances for success as very low (low self-confidence) and others’ ability as very high. This condition also existed during examination time.

“I'm slow at learning.” (Participant 10, female, failed twice)

“I can't pass the exam. I'm not ready for the exam.” (Participant 19, male, failed twice)

“I think that she has reviewed five times. How fortunate she is! She has improved, and surely she will be accepted this year.” (Participant 26, female, failed three times)

“It's enough to see the person next to me smile. My self-confidence drops to zero, and I tell myself that everything is finished. How fortunate she/he is!” (Participant 14, male, failed twice)

Two other subcategories were magnifying the difficulty of the examination and catastrophizing failure.

“This is the most difficult exam in the country. Other majors are not forced to participate in such exams that your entire life depends on.” (Participant 23, female, failed three times)

“I cannot bear other people’s looks. It is as if they are telling me that if you fail once more, for sure you will never pass again. Even my family has this feeling. If I fail, I will not be able to look them in the eye.” (Participant 7, male, failed four times)

The third subcategory, pessimism towards test designers, was accompanied by anger. Candidates believed that test designers intended to deceive them and designed questions in a way no one could answer. Over 90% of study participants shared this belief.

“For sure this year, they will give more difficult questions to humiliate us.” (Participant 11, male, failed twice)

Inner struggle included two subcategories related to the post-examination period. In the first, candidates asked themselves, “What will happen?” and were preoccupied with trying to predict their results. The second, sweet daydreaming, involved planning fun activities immediately after the examination. Sometimes, candidates were consumed by such thoughts.

“I wish there was a way to predict the exam result. I can’t tolerate this long waiting time until the day of the exam results.” (Participant 12, female, failed twice)

“When I’m studying for the exam, sometimes I look at the clock. I see that there is some time and I imagine the hours and days after the exam. I imagine myself free from everything, without any concern, traveling to north, having gotten an excellent grade on the exam.” (Participant 31, male, failed three times)

### Putting themselves under pressure

Exam candidates place themselves under immense pressure. This category included four subcategories. One of the common features of candidates was that they study long hours each day over the course of many months. Those who studied fewer hours believed they should have studied far more.

“I have been studying 14 hours a day for 15 months.” (Participant 7, male, failed four times)

“I study 13 hours each day but I don’t improve. I wish I could study more…” (Participant 18, male, failed twice)

The second subcategory, studying hard, manifested in behaviors such as reviewing too much and memorizing texts completely. Participants tended to memorize texts without understanding them.

“If you want to pass the exam, you should become like a computer where with a simple click, all references are present in your mind.” (Participant 25, female, failed three times)

The third subcategory consisted of participants who practised asceticism during the months prior to the exam. For example, some stopped going to parties, traveling, watching television and sports, and going on unpaid vacations. As a result, they were financially dependent on their spouse or family. Married participants frequently demonstrated behaviors such as putting their children in daycare or having their grandparents look after them.  During the exam, fruit juice and light snacks, such as cookies, are given to candidates to give them energy. However, some candidates refused to eat or drink during the examination, fearing that they would lose time. Some candidates did not even shift their position in their chair, saying that they would lose even a few seconds.

"Time should not be wasted. Drinking juice during the exam means losing time for one or two questions.” (Participant 19, male, failed twice)

Finally, most of the participants used doping to increase their output. This behavior was demonstrated in the consumption of methylphenidate, anti-anxiety drugs, strong coffee and tea, energy drinks, and foods with a high-calorie and high vitamin count.

### Inefficient mapping

“Inefficient mapping” refers to the inability to prioritize. This category was further broken down into three subcategories. After being confronted with unexpected questions on previous examinations, candidates were confused when attempting to select study material. In the first subcategory, homogenization, candidates were unable to prioritize and spent equal amounts of time on all study materials.

“Everything is important, even details and footnotes. If you look at my book, it is all marked and highlighted. Sometimes I think that it means that everything is important, so what purpose does highlighting serve?” (Participant 35, female, failed five times)

The next subcategory, excessive attention to detail, was seen in a group of candidates who, by wasting too much time memorizing minor details, spent less time on more critical materials. Sometimes, candidates revised highly specialised content in-depth, and yet forgot to review management of more basic medical conditions:

“Sometimes I think that I know everything in medicine. I know the details of chemotherapy of lymphoma, or I know the detailed receptors of HIV. But I don’t know the management of a simple flu. This exam has caused such ridiculousness. The designers like unimportant details.” (Participant 4, male, failed twice)

The third subcategory, searching for out-of-mind points, was reported by many candidates. In this case, candidates were intent on reading between the lines in order to discover hidden meanings in the textbooks, which often led to misinterpreting information.

“I have a notebook in which I have just written topics that I know but others don’t understand. If I had known them years ago, I would have passed the exam.” (Participant 21, female, failed four times)

### Improper final review

Candidates often experienced ambiguity and hesitation during examination time, which they attributed to inadequate review during the final days prior to the exam. The “Improper final review” category was divided into two subcategories. In the first, wasted review, some candidates simply believed that they did not need to review before the exam. This was accompanied by beliefs such as the need to rest, the futility of studying, and the need for fun in the days leading up to the exam. Some candidates said they worked hard to keep their minds busy with anything except the exam, so that they could sleep. Another group continued studying until the morning of the exam, leaving no time to review. 

Under the second subcategory, excessive review, participants reviewed in a furious, disorganized manner during the final days. One participant described her behavior metaphorically:

“In the days before the exam, I feel like someone who is trying to do everything to avoid drowning.” (Participant 21, female, failed four times)

### Afflicted body

Candidates complained of several body symptoms that they attributed to the time spent preparing for the exam. These problems peaked on examination day and were divided into three subcategories. The first was signs of anxiety, such as impatience, restlessness, confusion, repeated urinary urgency, low concentration, non-specific digestive signs, and headache. Some candidates had sweats and hand tremor.

“Sometimes, my heart beats so fast before the exam that if I don't take a propranolol pill, I can't take the exam.” (Participant 9, male, failed three times)

Under the second subcategory were signs related to fatigue, such as low energy, sleeplessness, and extreme tiredness. Weight gain, the third subcategory, was common in participants. More than 70% experienced weight gain preparing for the exam, which they attributed to lack of physical activity, eating as a form of relaxation, and lack of motivation and patience to take care of themselves.

“When I started to study 10 months ago, I was 70 kilograms. Now I am 80 kilograms. Eating is the only fun I have. When you live in a library, you can’t cook. You need to eat fast food, French fries, burgers, etc. There is also no time to exercise.” (Participant 17, female, failed three times)

### Ineffective test-taking skills

This category included six subcategories, the first of which was overcomplicating. According to the candidates, REE questions are complex. They considered each question a complex puzzle and expected important points to be hidden in them, thus continuously searching for these hidden meanings. They interpreted each question in different ways and expected the most complicated interpretation to be the most probable answer. They read questions obsessively, reading between the lines and prevailing over what they perceived to be the malicious intentions of test designers:

“When reading the question, I try to find hidden points in each because it is unlikely that a question has no specific point. There are few of these kinds of questions.” (Participant 20, female, failed twice)

The following subcategory, hastiness, involved carelessness reading the questions and rushing to answer. Some participants identified this behavior as a consequence of anxiety and fatigue; others believed that, if they thought about the question longer, they would choose the wrong answer more often.

“I don't read all the items. I choose the first correct item that appears in my mind.” (Participant 24, female, failed twice)

The third subcategory was answering questions in order. Participants insisted on answering the questions in the same order in which they were presented. This not only led to spending too much time on difficult questions, but it also caused confusion with the previous question while studying the next question.

“You should try hard to answer the questions in order unless you become tired and cannot anymore.” (Participant 10, female, failed twice)

The next subcategory was poor time management. In some cases, candidates spent more time answering questions at the beginning of the examination and less time at the end, or vice versa. In other cases, they spent too much time on easy questions and too little time on difficult ones.

In the fifth subcategory, hesitation at the end of the examination, some candidates not only hesitated to give answers at the end, but, in fact, began to change their answers, even though most of them believed it was detrimental to their results. Some candidates left the exam venue immediately after finishing, in order to prevent this temptation.

“When I reach the end [of the exam], a sense of deep doubt starts. I begin to change the answers on the answer sheet. I always know that the first idea is the best, but I can’t resist doing that.” (Participant 5, female, failed twice)

The sixth subcategory, obsessive checking, referred to candidates who obsessively checked their answer sheets in order to minimize errors entering responses, and thereby wasting a great deal of time.

### Liberation

The category of liberation referred to post-examination feelings in which candidates reported feeling as though they had been released from a hard labor camp. This category was divided into two subcategories. The first, happy liberation, referred to the feeling of indiscernible freedom that almost all candidates experienced. Before taking the exam, some candidates planned to travel on completion.

“I feel like a spring released from pressure.” (Participant 17, female, failed three times)

“The evening after the exam has another mode. It’s as if everything has changed, as if life has started again, and as if one can fly.” (Participant 23, female, failed three times)

Coexisting with happy liberation was the second subcategory, bitter liberation. In this case, candidates had mixed feelings of anger at test designers, aversion to the existing situation, regret at too little time to study, and anxiety about the final result.

“I really think that someone should like to torment others to design such questions. I would like to see them and beat [them] very much.” (Participant 11, male, failed three times)

## Discussion

To our knowledge, this is the first study to investigate the experiences of candidates preparing for a highly competitive examination despite having repeatedly failed it. We found two similar studies, but the focus was on other dimensions of failure, rather than on competition.^10^^,^^11^

Our study suggests that low performers in the REE place themselves under great pressure and use maladaptive strategies to manage their stress and to answer the questions. Here we attempted qualitatively to conceptualize this phenomenon. Consistent with the cognitive theory of anxiety,^27^ we suggest that, because candidates were fearful of failing, they perceived the REE as very difficult and repeat failure was probable. The expectation of success from their families also played a role in the anxiety of candidates, consistent with similar findings.^28^^-^^30^ This anxiety engendered two coping strategies: paranoia about the examination designers and sweet daydreaming. Some studies show that social stress, through decreased self-confidence, can create paranoid beliefs;^31^ especially in people with a history of anxiety.^32^ This represents the extreme aspect of attribution of failure to external factors reported by medical students who fail^10^ and plays a role in failure enhancement,^33^^,^^34^ especially when candidates believe that the competition is unfair.^35^ This contrasts with positive perceptions of and emotions toward the exam, both of which are associated with superior performance.^36^ As a coping mechanism, sweet daydreaming is employed to avoid the emotional pain of failure.^37^

Another way for candidates to increase their chances of success was by putting themselves under physical pressure with methylphenidate to decrease fatigue and increase concentration. In Iran, approximately 23% of residents misuse methylphenidate, the most important reason for which is participation in the REE.^38^ This contrasts starkly with the rate of use in other countries, approximately 5.3%.^39^ Methylphenidate can cause anxiety and fatigue.^40^ Therefore, participants subjected themselves to a vicious cycle of fatigue-doping, which is associated with declines in performance. Such pressure was also evident in inefficient mapping, a kind of non-adaptive perfectionism in attempting to master the material. Such perfectionism plays a role in overcompensating for the severe fear of failure^41^ and the lack of study skills common to most Iranian students.^42^ Moreover, those who have high examination anxiety usually have fewer study skills.^43^ These factors lead to severe stress, depression, low self-confidence, and, finally, a decline in performance.^44^ This problem is arguably more prominent in competitive high-level exams.

Some studies have demonstrated that leaving time to review before an examination can have positive effects on the candidate’s performance, especially if the review is executed in the form of a practice test (for example, the previous years’ examination), as opposed to a textbook review.^45^^-^^47^ However, there are various reasons to disregard reviewing. For example, it is human nature to overestimate one’s abilities^48^ and to develop an illusion of competence.^49^ This illusion may blind the candidate to the importance of reviewing study materials before the exam and lead to false trust in memory. On the other hand, excessive, furious, or unstructured reviewing negatively affects concentration and learning and will likely decrease performance.^50^

Symptoms of an afflicted body, owing to exhaustion and high stress levels were also common.^51^ Chronic stress is associated with elevated cortisol levels and heightened somatic arousal, which lead to physical, emotional, and cognitive burnout,^52^ creating an inefficient depreciation-stress cycle. Consistent with this explanation, weight gain is conceptualized as an emotion-focused coping strategy for high stress, e.g., eating food to relax.^53^

On examination day, to perfectionists and to those pessimistic about the designer’s intentions, the questions appeared not only complex, but also to contain hidden meanings. Students are usually able to alter their answering strategies according to the degree of difficulty of the questions.^54^^,^^55^ Furthermore, student predictions about the difficulty of the examination (test expectancy) has a direct effect on recall and recognition ability, and students perform according to these expectations.^56^^,^^57^ However, participants in the current study lacked such flexibility. They continued to answer until the end of the exam with anxiety, without the ability to process opposing information. This topic is similar to schemata information processing, in which the mind evaluates all observations received via schematic filters, confirms the schemata, and ignores irrelevant information.^58^ Therefore, repeated failures can lead to the formation of the schemata, such as, “The exam is difficult,” “The designers are the enemy,” and “I can't pass the exam.” Such schemata lead to the prediction of difficulty before the examination and to interpreting questions as difficult during the examination. The result is anxiety and maladaptive strategies to decrease such anxiety, such as answering in order, hesitating too much at the end of the examination, obsessively checking the answer sheet, and answering hastily. Such strategies are clearly detrimental to candidate performance. Poor time management also complicated the issue. Further investigations are required to investigate this hypothesis. If confirmed, interventional planning could greatly reduce anxiety and thus improve performance.

The final finding, liberation, is unique, and has not been referred to in previous, related studies. These feelings might indicate the intense pressure felt by examination candidates. In recent research, attention has been focused on emotions such as the anger, disappointment, and shame felt by test takers with regard to their performance.^59^ These emotions directly affect motivation, material learning strategies, self-regulation, and educational success. In this area, there is a model that believes the educational objectives of the individual engender these emotions, and that these emotions predict their success.^60^^,^^61^ More broadly, these emotions mediate different variables affecting educational function.^62^^,^^63^ Therefore, one important function of these emotions is to form the motivation for studying and, consequently, degree of success on the following year’s exam.

In summary, this study shows that REE exerts great pressure on participants with repeated failure. It cannot be concluded, however, that these participants are not right candidates for medical universities. REE assesses just the intellectual ability of candidates, and no other important factors such as learning styles, communication ability and personality that are important to select students for medical courses. Studies show that an optimum combination of these factors should be considered to select medical students for medical universities, and being on the extreme side of high intellectual ability is not enough to select a candidate for medical education.^64^ On the other hand, performance on the entrance exam is a relatively poor predictor of future academic performance, and that its predictive validity declines over the academic years of medical school.^65^ So, it can be hypothesized that the two following approaches might be useful to manage the condition. First, some interventions can be used to decrease burnout and chance of failure, for example: teaching study skills,^66^^,^^67^ cognitive-behavioral stress management, interventions to improve self-efficacy, forming self-assistance groups for examination candidates, teaching mindfulness, relaxation, and providing advice, for example on body activity enhancement.^68^^,^^69^ Second, this study raises the concern surrounding the usefulness of ‘one size fits all’ policy for university entrance exams, and the usefulness of one-dimensional assessment. So, policy makers need to consider other kinds of assessment to improve the quality of medical university entrance exams.

There are several limitations to this study. First, it cannot be concluded whether these reactions are temporary and secondary to the REE or whether they represent a collection of individual characteristics, pronounced when confronted by the REE. Second, the experience of those candidates who pass the REE the first or second time is unrecognized here. Further research is necessary to explore the experiences of those candidates. Moreover, although sampling was performed from a university library from among a variety of different physicians, this library was not representative of all libraries in the country. Lastly, the lack of assessment of previous educational level, predisposing factors to educational failure, social background^70^ and specialty preferences are further limitations.

## Conclusions

Candidates who failed the REE pressured themselves for 12 to 18 months to participate in the examination the following year. They were confused, anxious, and pessimistic. They were unable to prioritize study materials and reviewed improperly. They entered the exam with an afflicted body and answered the questions anxiously, inefficiently, hastily, and obsessively. Finally, they experienced the feeling of having escaped from prison, in the context of both negative and positive emotions. As emotion-focused coping strategies, all these increase perceived levels of stress^71^ and subject participants to a vicious cycle of stress. These experiences may be considered a reaction to the REE as a highly-competitive examination with far-reaching consequences for the social and economic conditions of general practitioners in Iran, where wide variety of individual, social, and biological factors exist. Because this climate has a negative impact on students learning styles, well-being and educational satisfaction, there is a need to develop new curriculums for prospective specialists in Iran.

### Acknowledgements

We acknowledge the staff of Rasoul-e-Akram Hospital for their cooperation in providing an appropriate environment for the interviews.

### Conflict of Interest

The authors declare that they have no conflict of interest.
